# Effects of Dry-Cured Ham Consumption on Cardiometabolic and Vascular Health in Adults: A Systematic Review and Meta-Analysis of Human Intervention Studies

**DOI:** 10.3390/foods15071198

**Published:** 2026-04-02

**Authors:** Manuel Hernández-Lorca, Desirée Victoria-Montesinos, Ana María García-Muñoz, Eva Salazar, Adela Abellán

**Affiliations:** 1 Department of Nutrition and Food Technology, Universidad Católica de Murcia-UCAM, Campus de los Jerónimos, 30107 Murcia, Spain; mhernandez2@ucam.edu (M.H.-L.); esalazar@ucam.edu (E.S.); aabellan@ucam.edu (A.A.); 2 Faculty of Pharmacy and Nutrition, UCAM Universidad Católica de Murcia, 30107 Murcia, Spain

**Keywords:** dry-cured ham, processed meat, cardiometabolic health, blood pressure, lipid profile, food matrix, meta-analysis

## Abstract

Cardiovascular disease is a leading cause of global morbidity and mortality, and processed meat consumption has been consistently associated with adverse cardiometabolic outcomes in observational studies. However, processed meat products differ substantially in composition and processing methods, and traditional dry-cured ham presents distinct nutritional and biochemical characteristics. This systematic review and meta-analysis aimed to synthesize evidence from human intervention studies evaluating the effects of dry-cured ham consumption on cardiometabolic and vascular health in adults. A comprehensive search of major databases identified eligible randomized and non-randomized intervention studies. Five trials were included in the qualitative synthesis, and meta-analyses were performed for blood pressure, lipid profile, and fasting blood glucose outcomes when sufficient data were available. The pooled analyses indicated a small but statistically significant reduction in diastolic blood pressure and total cholesterol associated with dry-cured ham consumption, whereas no significant effects were observed for systolic blood pressure, LDL cholesterol, HDL cholesterol, triglycerides, or fasting blood glucose. Substantial heterogeneity was present across most outcomes. Overall, the available intervention evidence suggests that dry-cured ham consumption at doses ranging from 40 to 120 g/day does not appear to adversely affect conventional cardiometabolic risk markers in adults. Nevertheless, the limited number and short duration of trials warrant cautious interpretation.

## 1. Introduction

Cardiovascular disease (CVD) represents the leading cause of mortality and disability worldwide and constitutes a major global public health challenge. According to the Global Burden of Disease (GBD) 2019 study, CVD accounts for a substantial proportion of premature deaths and years lived with disability, with cardiometabolic risk factors such as elevated blood pressure, dyslipidemia, impaired glucose metabolism and chronic low-grade inflammation playing a central role in its etiology and progression [1]. Importantly, GBD analyses have consistently identified dietary risk factors as major contributors to cardiometabolic morbidity and mortality across regions and populations, highlighting diet as a cornerstone in both prevention and risk-reduction strategies [2,3].

Within this context, processed meat consumption has been repeatedly examined in epidemiological research and evidence syntheses, with several systematic reviews and meta-analyses reporting associations between higher processed meat intake and increased risk of cardiovascular disease, type 2 diabetes and all-cause mortality [4,5]. For example, in pooled cohort analyses, each additional two servings of processed meat per week was associated with a 7% higher risk of incident cardiovascular disease [5]. These associations have informed dietary guidelines that generally recommend limiting processed meat intake as part of cardiometabolic disease prevention. Nevertheless, some reviews have highlighted that the health effects traditionally attributed to processed or red meat may be partly confounded by substantial heterogeneity in meat composition rather than meat intake alone. For instance, Smith et al. [6] reported that interventions using high-oleic acid ground beef did not increase cardiovascular disease risk factors and, in several human trials, were associated with increases in high-density lipoprotein cholesterol, comparable to those observed with high-oleic acid oils. These findings suggest that differences in fatty acid profiles, particularly in the relative content of oleic acid, saturated fatty acids and trans fatty acids, may contribute to the inconsistent associations observed across epidemiological studies.

Importantly, the broad classification of processed meats encompasses products with markedly different production methods, food matrices and nutritional profiles. Traditional dry-cured hams, such as Spanish serrano and Iberian ham, differ substantially from industrially processed meats in terms of curing duration, absence of thermal processing and endogenous biochemical changes occurring during maturation [7]. Iberian ham is obtained from the autochthonous Iberian pig (*Sus scrofa*), whereas serrano ham is produced from commercial white pig crosses and manufactured through traditional salting and long-term dry-curing processes recognized under the European Union Protected Geographical Indication quality scheme [8]. Reviews focusing on meat processing and structure have emphasized that prolonged curing promotes proteolysis and peptide generation while preserving the integrity of the muscle food matrix, potentially resulting in physiological effects distinct from those of restructured or highly processed meat products [7,9]. These characteristics suggest that treating dry-cured ham as nutritionally equivalent to other processed meats may obscure relevant food-specific effects.

A further distinguishing feature of dry-cured ham is its lipid composition, particularly the relatively high proportion of monounsaturated fatty acids, with oleic acid being the predominant fatty acid. Evidence from systematic reviews and meta-analyses indicates that dietary patterns richer in monounsaturated fatty acids and the substitution of saturated fatty acids with unsaturated fats are generally associated with more favorable cardiometabolic risk profiles [10,11]. This pattern has been observed particularly for lipid parameters and other cardiovascular risk markers. These findings provide biological plausibility for the differential cardiometabolic effects of dry-cured ham compared with processed meats characterised by higher saturated fat content.

Beyond its fatty acid profile, dry-cured ham has gained increasing attention as a source of bioactive peptides generated during the curing process through endogenous enzymatic activity. Recent comprehensive reviews have described the presence of peptides with potential angiotensin-converting enzyme inhibitory, antioxidant and endothelial-modulating properties in dry-cured ham, supporting mechanistic pathways relevant to blood pressure regulation and vascular health [7,9]. This emerging body of evidence reinforces the need to consider dry-cured ham not merely as a source of sodium or fat, but as a complex food matrix with potential functional properties. However, the human intervention studies included in the present review did not directly quantify circulating ham-derived peptides or assess their in vivo activity after consumption.

In parallel, advances in processing technology have led to the development of dry-cured hams with reduced nitrate and nitrite content, addressing longstanding concerns regarding the formation of N-nitroso compounds [12,13]. Contemporary reviews of nitrite and nitrate use in meat processing highlight ongoing reformulation strategies aimed at reducing additive exposure while maintaining microbiological safety and product quality, particularly in traditionally cured products [14]. These developments further contribute to the evolving nutritional profile of dry-cured ham and may influence its cardiometabolic and vascular effects.

Despite these distinctive characteristics, most observational studies and meta-analyses continue to aggregate dry-cured ham with other processed meat products, potentially masking food-specific associations [15]. In contrast, several human studies have directly evaluated dry-cured ham consumption under controlled dietary conditions, reporting neutral or potentially beneficial effects on cardiometabolic and vascular outcomes such as blood pressure, lipid profile and endothelial function [16,17]. However, the available evidence remains fragmented and heterogeneous and has not yet been systematically synthesised with a specific focus on dry-cured ham as a whole food.

In this context, a systematic review enables the available intervention studies to be examined in a structured and transparent way, taking into account differences in study design, comparator foods, intervention duration, and outcomes assessed. When possible, meta-analysis adds a quantitative component by integrating results across studies and helping to determine whether the observed findings point in a consistent direction. This approach is especially relevant here, where the evidence remains limited and scattered across a small number of human studies.

To our knowledge, no systematic review and meta-analysis has specifically synthesized human intervention studies focusing on dry-cured ham consumption. Therefore, the present systematic review aims to evaluate the effects of dry-cured ham consumption on cardiometabolic and vascular markers in adults, including blood pressure and lipid profile as primary outcomes and endothelial function, inflammatory biomarkers, and oxidative stress as secondary outcomes.

## 2. Materials and Methods

### 2.1. Study Design and Protocol Registration

This systematic review was designed and conducted in accordance with the Preferred Reporting Items for Systematic Reviews and Meta-Analyses (PRISMA) 2020 guidelines [18]. A predefined protocol describing the objectives, eligibility criteria, outcomes, and planned analyses was prospectively registered in the International Prospective Register of Systematic Reviews (PROSPERO) under the registration number CRD420261321334.

### 2.2. Eligibility Criteria

The present review aimed to synthesize the available evidence from human intervention studies evaluating the health effects of cured ham consumption. Eligibility criteria were defined a priori according to the PICOS framework [19], which specified the following elements: Population, adults aged 18 years or older, including healthy individuals and those with mild cardiometabolic risk factors; Intervention, consumption of cured ham as a whole food, including dry-cured varieties such as Iberian ham or Spanish serrano ham; Comparator, usual diet, diets excluding cured ham, substitution with other meat or protein products, or baseline values in non-randomized studies; Outcomes, cardiometabolic and vascular health markers; and Study design, randomized controlled trials and non-randomized prospective intervention studies.

Eligible participants were adults aged 18 years or older, including healthy individuals and adults presenting mild cardiometabolic risk factors, such as prehypertension, borderline hypertension, mild dyslipidemia, or low to moderate cardiovascular risk. Studies conducted in community-dwelling adults, older populations, or primary care settings were considered eligible, provided that participants did not present established cardiovascular disease or severe chronic conditions at baseline.

The intervention of interest was the consumption of cured ham, including dry-cured ham varieties, such as Iberian ham or Spanish serrano ham, administered as part of the habitual diet and consumed as a whole food. Studies evaluating cured ham within controlled dietary interventions, regardless of dose or duration, were eligible. Interventions based on isolated compounds, supplements, or extracts derived from cured ham were excluded.

Eligible comparators included usual diet, diets excluding cured ham, or substitution with other protein or meat products (e.g., cooked ham or fresh meat). In non-randomized intervention studies, baseline values prior to cured ham consumption were accepted as comparators.

The outcomes of interest included cardiometabolic and vascular health markers, such as blood pressure, lipid profile parameters, endothelial function, inflammatory biomarkers, oxidative stress markers, and global cardiovascular risk scores. Studies were required to report at least one relevant health-related outcome measured before and after the intervention.

Randomized controlled trials (parallel or crossover designs) and non-randomized prospective intervention studies were eligible for inclusion. Observational studies without dietary intervention, cross-sectional studies, narrative reviews, editorials, conference abstracts, animal studies, and in vitro studies were excluded.

### 2.3. Information Sources and Search Strategy

A comprehensive literature search was performed in the electronic databases PubMed/MEDLINE, Web of Science, Scopus, and the Cochrane Central Register of Controlled Trials (CENTRAL) from inception to the most recent search date. The search strategy combined controlled vocabulary terms and free-text keywords related to cured ham consumption and health outcomes. The complete search strategies for all databases are detailed in Table S1.

Search terms included combinations of the following concepts: “cured ham”, “dry-cured ham”, “Iberian ham”, “jamón serrano” combined with terms related to cardiovascular, metabolic, inflammatory, and oxidative stress outcomes, such as “blood pressure”, “hypertension”, “endothelial function”, “lipid profile”, “cardiovascular risk”, “inflammation”, and “oxidative stress”. Search strategies were adapted to the syntax and indexing terms of each database.

In addition, the reference lists of all included articles and relevant reviews were manually screened to identify any additional eligible studies not captured through the electronic search.

### 2.4. Study Selection

All records retrieved from the database searches were imported into reference management software, and duplicate entries were removed. Two reviewers (D.V.-M. and A.M.G.-M.) independently screened titles and abstracts to assess eligibility. Studies considered potentially relevant were retrieved in full text and evaluated against the predefined inclusion and exclusion criteria.

Discrepancies between reviewers at any stage of the selection process were resolved through discussion and consensus. When agreement could not be reached, a third reviewer was consulted.

### 2.5. Data Extraction

Data extraction was performed independently by one reviewer (M.H.-L.) and subsequently verified by a second reviewer to ensure accuracy (D.V.-M.). The following information was extracted from each included study: author and year of publication, country, study design, sample size, participant characteristics, details of the intervention (type of cured ham, dose, frequency, and duration), comparator characteristics, outcomes assessed, methods of outcome measurement, and main results.

When required data were unclear or not fully reported, attempts were made to infer values from the published data or Supplementary Materials.

### 2.6. Risk of Bias Assessment

The methodological quality and risk of bias of the included studies were assessed using validated tools according to study design. Randomized controlled trials were evaluated using the Cochrane Risk of Bias tool (RoB 2.0) [20,21], which assesses potential sources of bias across predefined domains, including bias arising from the randomization process, deviations from intended interventions, missing outcome data, measurement of the outcome, and selection of the reported result. Each domain was judged as presenting a low risk of bias, some concerns, or a high risk of bias, and an overall risk of bias judgment was assigned to each trial.

For non-randomized intervention studies, the Risk Of Bias In Non-randomized Studies of Interventions (ROBINS-I) tool was applied [22,23]. This tool evaluates bias due to confounding, selection of participants, classification of interventions, deviations from intended interventions, missing data, measurement of outcomes, and selection of the reported result, allowing a structured assessment of internal validity in the absence of randomization.

Risk of bias assessments were conducted independently by two reviewers (D.V.-M. and A.M.G.-M.). Any discrepancies in judgments were resolved through discussion and consensus, and when necessary, consultation with a third reviewer (M.H.-L.) was undertaken to reach agreement.

### 2.7. Data Synthesis and Statistical Analysis

A formal data synthesis was planned. Quantitative synthesis (meta-analysis) was conducted when at least two randomized controlled trials reported comparable outcomes. Meta-analyses were performed for lipid profile, fasting blood glucose, and blood pressure parameters when sufficient data were available.

For continuous outcomes, pooled effect estimates were calculated using mean differences (MDs) when identical measurement methods were used across studies, or standardized mean differences (SMDs) when potential differences in measurement methods could not be ruled out, with corresponding 95% confidence intervals (CIs).

For parallel-group trials, treatment effects were calculated as the difference in pre-post changes between intervention and control groups. For crossover trials, treatment effects were preferentially extracted from the authors’ adjusted models, accounting for period and sequence effects. When such adjusted estimates were not available, effects were derived from pre-post change scores by intervention and treated analytically as parallel comparisons.

For outcomes requiring estimation of the variance of change scores, within-subject correlations were taken into account. In the absence of reported correlation coefficients, a within-subject correlation coefficient of r = 0.8 was assumed in the primary analysis. Sensitivity analyses using alternative correlation assumptions (r = 0.5) were conducted to assess the robustness of the findings.

Statistical heterogeneity was assessed using Cochran’s Q test and quantified using the I^2^ statistic. Random-effects models were applied to account for between-study heterogeneity.

Outcomes not suitable for meta-analysis because of heterogeneity in outcome definitions, measurement methods, or a limited number of studies (e.g., endothelial function, inflammatory biomarkers, oxidative stress markers, and cardiovascular risk scores) were synthesized narratively using a structured qualitative approach.

Statistical analyses were performed using Stata software (version 19.5; StataCorp, College Station, TX, USA), and statistical significance was set at *p* < 0.05.

## 3. Results

### 3.1. Study Selection

**Figure 1:**
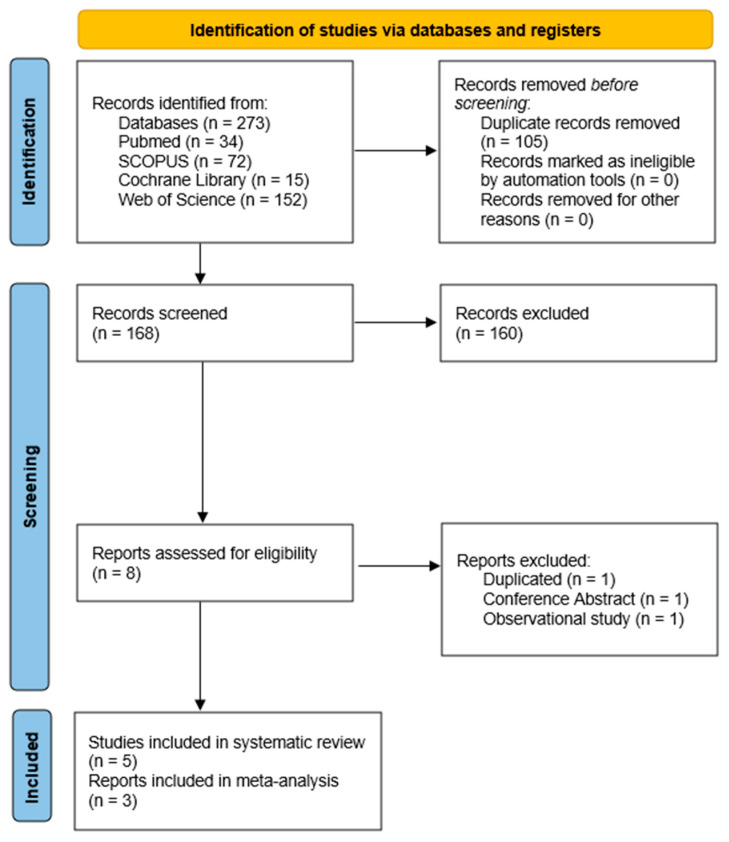
PRISMA 2020 flow diagram of the study selection process.

A total of eight full-text articles were assessed for eligibility. Following detailed evaluation, five clinical trials [24,25,26,27,28] met the predefined inclusion criteria and were included in the qualitative synthesis (Figure 1). Two publications [16,25] derived from the same randomized crossover cohort (BACCHUS project) were integrated to avoid double-counting of participants.

The included studies were published between 2003 and 2022 and comprised randomized controlled trials (parallel and crossover designs), one prospective quasi-experimental study, and one non-randomized dietary intervention. After full-text assessment, several studies were excluded for specific reasons: one publication by Montoro-García et al. was excluded because it was published solely as a conference communication [29], another study by Montoro-García et al. [16] was excluded due to duplication of the study population, and the study by Mora et al. [30] was excluded because it did not involve a dietary intervention.

### 3.2. Study Characteristics

Across the five included intervention studies, a total of 315 participants were enrolled. Sample sizes ranged from 21 to 102 individuals. The pooled, sample-size-weighted mean age was 45 years, although one study specifically targeted older adults with a mean age of approximately 71 years. Sex distribution varied substantially across studies, with female participation ranging from 18% to 77%. Overall, women represented approximately 49% of participants in studies reporting sex distribution.

All studies were conducted in Spain. The interventions evaluated Iberian dry-cured ham obtained from pigs raised under different feeding systems (acorn-fed or feed-fed) and consumed as a whole food, with daily doses ranging from 40 g to 120 g. Intervention duration ranged from acute 4-week crossover periods to 8-week parallel interventions.

Three studies were randomized controlled trials (two crossover and one parallel) [24,25,27], one was a prospective quasi-experimental intervention [28], and one was a non-randomized dietary substitution study in older adults [26].

The main characteristics of the included studies are summarized in Table 1.

### 3.3. Effects of Dry-Cured Ham on Cardiometabolic and Vascular Outcomes

#### 3.3.1. Blood Pressure and Hemodynamic Parameters

Four studies evaluated blood pressure outcomes. In the BACCHUS crossover trial [25], consumption of 80 g/day of dry-cured ham rich in bioactive peptides for four weeks did not adversely affect office blood pressure or 24-h urinary sodium excretion compared with cooked ham. No clinically relevant increases in systolic or diastolic blood pressure were observed, suggesting that moderate intake of dry-cured ham did not adversely affect blood pressure in prehypertensive individuals. In contrast, Montoro-García et al. [24] reported significant reductions in 24-h ambulatory systolic and diastolic blood pressure during the dry-cured ham intervention period compared with the control (cooked ham) period in adults with cardiometabolic risk factors. The magnitude of reduction was modest but statistically significant and was observed in both daytime and nighttime measurements.

Mayoral et al. [26], in a non-randomized intervention among older adults, reported a significant reduction in mean arterial pressure during the six-week ham substitution period compared with the basal diet phase. Although the small sample size and non-randomized design limit internal validity, these findings suggest potential hemodynamic benefits in older populations. Finally, Saban-Ruiz et al. [27] did not observe significant changes in clinical blood pressure values in healthy adults following six weeks of Iberian cured-ham consumption.

Taken together, the evidence suggests that moderate consumption of dry-cured Iberian ham does not worsen blood pressure and may be associated with modest reductions in ambulatory or mean arterial pressure in specific populations.

**Table 1:** Characteristics of the included studies.

Author (Year)	Country	Study Design	Duration	Population	N (% Female)	Age (Years)	Intervention	Comparator	Outcomes	Biological Sample
Mayoral et al. (2003) [26]	Spain	Non-randomized, open-label, single-arm pre-post intervention (sequential reversal design)	6 weeks ham diet + 6 weeks post-intervention	Older adults	21 (38.1)	71	Substitution of 120 g/day meat with acorn-fed Iberian ham (120 g/day)	Basal diet (before/after)	Mean arterial pressure (MAP); total antioxidant substances (TAS); glutathione reductase (GR); glutathione peroxidase (GPx); superoxide dismutase (SOD); lipid peroxidation (thiobarbituric acid reactive substances, TBARS)	Blood (plasma and erythrocyte-related markers)
Márquez-Contreras et al. (2018) [28]	Spain	Prospective quasi-experimental (pre-post)	8 weeks	Healthy adults without CVD/diabetes	100 (64)	42.1 ± 9.6	100% acorn-fed Iberian ham, 40 g/day	Baseline/habitual diet (pre-intervention)	SCORE cardiovascular risk; lipid profile (TC, LDL-c, HDL-c, TG); blood pressure; anthropometry; inflammation-related biochemistry (e.g., CRP)	Fasting blood biochemistry + clinical measures
Martínez-Sánchez et al. (2017) [25]	Spain	Randomized, placebo-controlled, crossover trial	4 weeks + 2-week washout + 4 weeks	Adults with elevated blood pressure (SBP > 125 mmHg)	38 (18.0)	44.3 ± 5.3	Dry-cured ham rich in bioactive peptides, 80 g/day	Cooked ham, 100 g/day	Blood pressure; 24 h sodium excretion; lipid profile and fasting glucose; inflammatory biomarkers (e.g., IL-6, MCP-1); platelet activation (P-selectin, PAC-1 after ADP stimulation); monocyte activation markers by flow cytometry	Venous blood (plasma ELISA); whole blood flow cytometry (platelet/monocyte markers)
Montoro-García et al. (2022) [24]	Spain	Randomized, placebo-controlled, crossover trial	28 days + 2-week washout + 28 days	Adults with normal-to-high blood pressure or mild cardiometabolic risk	54 (35.0)	49.0 ± 10.3	Dry-cured ham, 80 g/day (>12 months proteolysis)	Cooked ham, 100 g/day	24 h ambulatory BP (SBP/DBP day and night); lipid profile; fasting glucose/insulin-related outcomes (including HOMA-IR); appetite hormones (ghrelin, leptin)	Blood (fasting biochemistry/hormones) + ambulatory BP monitoring
Saban-Ruiz et al. (2017) [27]	Spain	Randomized, open-label, parallel trial	6 weeks (+6-week follow-up in intervention group)	Healthy adults	102 (76.8)	40.2 ± 8.7	Iberian cured-ham, 50 g/day	Diet without Iberian cured-ham	Endothelial function biomarkers (including plasminogen activator inhibitor-1, PAI-1); microvascular vasodilatory response to hyperemia; arterial stiffness; clinical parameters	Blood (endothelial/oxidative markers) and vascular function testing

Abbreviations: ADP (adenosine diphosphate), BP (blood pressure), CRP (C-reactive protein), ELISA (enzyme-linked immunosorbent assay), GR/GPx/SOD (glutathione reductase/glutathione peroxidase/superoxide dismutase), HDL-c (high-density lipoprotein cholesterol), HOMA-IR (homeostasis model assessment of insulin resistance), LDL-c (low-density lipoprotein cholesterol), MAP (mean arterial pressure), SBP/DBP (systolic/diastolic blood pressure), TAS (total antioxidant substances), TBARS (thiobarbituric acid reactive substances), TC (total cholesterol), TG (triglycerides).

#### 3.3.2. Lipid Profile and Cardiometabolic Markers

Three studies assessed lipid outcomes. Márquez-Contreras et al. [28], in a prospective quasi-experimental study of 100 healthy adults, reported improvements in lipid parameters after eight weeks of consuming 40 g/day of acorn-fed Iberian ham. Specifically, high-density lipoprotein cholesterol (HDL) increased, while low-density lipoprotein cholesterol (LDL) and triglycerides decreased. No significant changes were observed in global cardiovascular risk (SCORE), body weight, or blood pressure.

In the BACCHUS crossover trial [25], total cholesterol and LDL cholesterol decreased following the dry-cured ham intervention compared with the cooked ham control, and basal glucose levels were modestly reduced.

Similarly, Montoro-García et al. [24] observed a reduction in total cholesterol during the dry-cured ham period compared with the cooked ham period in individuals with cardiometabolic risk. However, no significant differences were found for fasting glucose, insulin, or HOMA-IR between treatment periods.

Overall, available data indicate that moderate dry-cured ham consumption does not adversely affect lipid parameters. Across individual studies, favorable changes were reported for total cholesterol, LDL cholesterol, HDL cholesterol, and triglycerides, although these findings were not fully consistent across trials.

#### 3.3.3. Endothelial Function and Vascular Biomarkers

Endothelial and vascular outcomes were primarily evaluated in Saban-Ruiz et al. [27]. In this randomized parallel trial, six weeks of Iberian cured-ham consumption resulted in a significant reduction in plasminogen activator inhibitor-1 (PAI-1), a biomarker associated with endothelial dysfunction. Additionally, microvascular vasodilatory response to hyperemia improved in the intervention group compared with controls. These findings suggest a favorable effect on early vascular function markers.

In the BACCHUS cohort [25], inflammatory and platelet activation markers were assessed. Dry-cured ham consumption was associated with reductions in certain inflammatory cytokines, including MCP-1 and IL-6 (borderline in some analyses), as well as reduced platelet activation following ADP stimulation. Changes in monocyte activation markers were also reported, indicating potential modulation of immune-endothelial interactions.

Collectively, these findings suggest that dry-cured ham may exert biological effects beyond conventional lipid parameters, potentially influencing endothelial function and inflammatory pathways.

#### 3.3.4. Oxidative Stress and Antioxidant Status

Mayoral et al. [26] evaluated oxidative stress markers in older adults and reported increases in total antioxidant substances and antioxidant enzyme activities (including superoxide dismutase, glutathione peroxidase, and glutathione reductase) during the ham intervention period. Concurrently, plasma lipid peroxidation markers (TBARS) decreased.

Although derived from a small, non-randomized study, these findings support the hypothesis that acorn-fed Iberian ham may influence oxidative stress balance, possibly owing to its lipid composition and other bioactive characteristics. No other included trials directly assessed systemic oxidative stress biomarkers.

### 3.4. Quantitative Synthesis (Meta-Analysis)

#### 3.4.1. Effects on Lipid Profile

**Figure 2:**
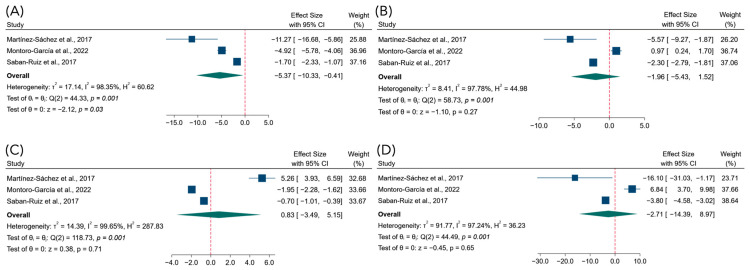
Forest plots of the effects of the intervention on lipid profile parameters: (**A**) Total cholesterol [24,25,27]; (**B**) low-density lipoprotein cholesterol [24,25,27]; (**C**) high-density lipoprotein cholesterol [24,25,27]; and (**D**) triglycerides [24,25,27]. Effect estimates are expressed as mean differences with 95% confidence intervals.

The pooled analysis showed a statistically significant reduction in total cholesterol levels in the intervention group compared with the control group (Figure 2A), with a mean difference of −5.37 mg/dL (95% CI: −10.33 to −0.41; *p* < 0.03). Between-study heterogeneity was observed (τ^2^ = 17.14; I^2^ = 98.35%; *p* < 0.001). For LDL, the pooled effect estimate did not indicate a statistically significant difference between intervention and control groups (Figure 2B), as the mean difference was −1.96 mg/dL (95% CI: −5.43 to 1.52; *p* = 0.27), with heterogeneity observed across studies (τ^2^ = 8.41; I^2^ = 97.78%; *p* < 0.001). No statistically significant differences were observed for HDL (Figure 2C), with a pooled mean difference of 0.83 mg/dL (95% CI: −3.49 to 5.15; *p* = 0.71; τ^2^ = 14.39; I^2^ = 99.65%; *p* < 0.001). Likewise, the meta-analysis did not demonstrate a significant effect of the intervention on triglyceride levels (Figure 2D). The pooled mean difference was −2.71 mg/dL (95% CI: −14.39 to 8.97; *p* = 0.65), with heterogeneity observed (τ^2^ = 91.77; I^2^ = 97.24%; *p* < 0.001).

Sensitivity analyses using an alternative within-subject correlation assumption (r = 0.5) yielded results consistent with the primary analyses, with no relevant changes in the direction or statistical significance of the pooled estimates for lipid outcomes (Supplementary Figure S1).

#### 3.4.2. Effects on Blood Pressure

**Figure 3:**
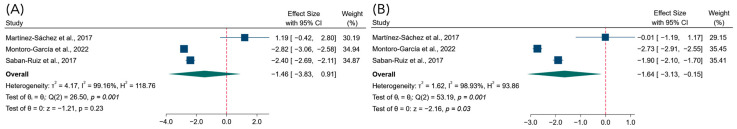
Forest plots of the effects of the intervention on blood pressure parameters. (**A**) Systolic blood pressure [24,25,27] and (**B**) diastolic blood pressure [24,25,27]. Effect estimates are expressed as mean differences with 95% confidence intervals.

The pooled analysis did not show a statistically significant effect of the intervention on systolic blood pressure compared with the control group (Figure 3A). The overall mean difference was −1.46 mmHg (95% CI: −3.83 to 0.91; *p* = 0.23), and between-study heterogeneity was observed (τ^2^ = 4.17; I^2^ = 99.16%; *p* < 0.001). In contrast, the pooled analysis for diastolic blood pressure indicated a statistically significant reduction associated with the intervention compared with the control group (Figure 3B), with a mean difference of −1.64 mmHg (95% CI: −3.13 to −0.15; *p* < 0.03). Heterogeneity was observed across studies (τ^2^ = 1.62; I^2^ = 98.93%; *p* < 0.001). Sensitivity analyses using an alternative within-subject correlation coefficient yielded results comparable to those of the primary analyses, with no change in the overall conclusions (Supplementary Figure S2).

#### 3.4.3. Effect on Fasting Blood Glucose

**Figure 4:**
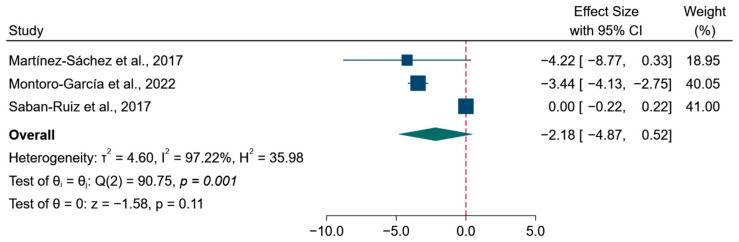
Forest plot of the effects of the intervention on fasting blood glucose [24,25,27]. Effect estimates are expressed as mean differences with 95% confidence intervals and were pooled using random-effects models.

For fasting blood glucose levels, the pooled analysis did not show a statistically significant effect of the intervention compared with the control group (Figure 4). The estimated mean difference was −2.18 mg/dL (95% CI: −4.87 to 0.52; *p* = 0.11), with heterogeneity across studies (τ^2^ = 4.60; I^2^ = 97.22%; *p* < 0.001). Applying an alternative within-subject correlation assumption yielded results comparable to those of the primary analysis. The pooled effect estimate remained virtually unchanged (mean difference: −2.17 mg/dL, 95% CI: −4.87 to 0.53; *p* = 0.11), with no relevant changes in the direction or statistical significance of the findings (Supplementary Figure S3).

### 3.5. Risk of Bias

**Figure 5:**
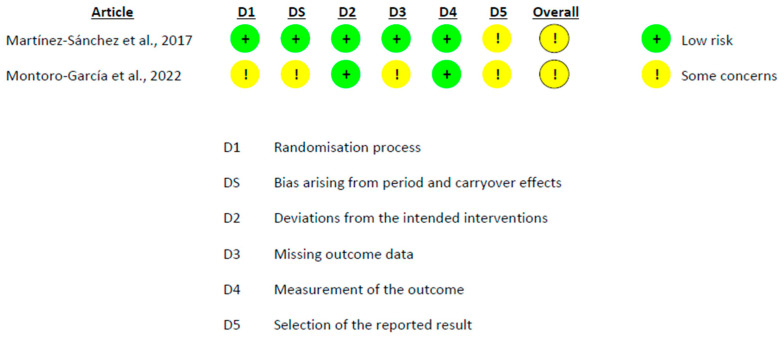
Risk of bias assessment of crossover randomized controlled trials using the RoB 2 tool [24,25].

**Figure 6:**
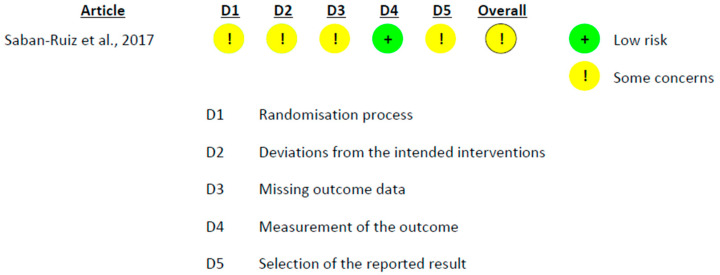
Risk of bias assessment of parallel-group randomized controlled trials using the RoB 2 tool [27].

The methodological quality of the included RCTs was assessed using the RoB 2 tool, applying the version specific to parallel-group or crossover designs, as appropriate. Overall, all included RCTs were judged as having *some concerns* regarding risk of bias (Figure 5 and Figure 6). In the crossover trials, the main source of uncertainty was related to selective reporting, as a fully accessible study registration or pre-specified analysis protocol was not available. In contrast, the remaining domains, including the randomization process, deviations from the intended interventions, missing outcome data, and outcome measurement, were generally judged to be at low risk of bias. In the parallel-group trial, additional concerns arose from the open-label design and the lack of explicit reporting of intention-to-treat analyses, although no critical sources of bias were identified.

**Figure 7:**
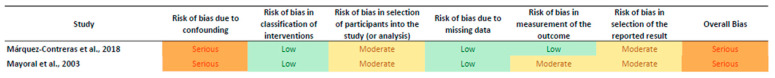
Risk of bias assessment of non-randomized intervention studies using the ROBINS-I tool [26,28].

The methodological quality of non-randomized intervention studies was evaluated using the ROBINS-I tool. Both non-randomized studies were judged to be at serious risk of bias overall (Figure 7), primarily driven by confounding inherent to their uncontrolled designs. In particular, the absence of a concurrent control group and the potential influence of time-related factors limited causal inference. Despite these limitations, the studies provided complementary evidence and were therefore retained, with their findings interpreted cautiously.

## 4. Discussion

This systematic review and meta-analysis synthesized evidence from five human intervention studies evaluating the effects of dry-cured ham consumption on cardiometabolic and vascular health in adults. Overall, the quantitative synthesis indicated a small but statistically significant reduction in diastolic blood pressure and a modest decrease in total cholesterol, while no significant effects were observed for LDL cholesterol, HDL cholesterol, triglycerides, or fasting blood glucose when compared with control interventions. Given the small magnitude of the observed effects and the substantial between-study heterogeneity, these findings should be interpreted with caution. Nonetheless, the available evidence suggests that moderate consumption of Iberian dry-cured ham does not adversely affect conventional cardiometabolic risk markers and may be associated with modest improvements in selected hemodynamic and lipid parameters in specific adult populations.

From a broader perspective, these findings differ from the adverse associations generally reported for processed meat consumption in large observational cohort studies, in which higher intakes of processed or red meat have been linked to increased risks of cardiovascular disease, type 2 diabetes, and hypertension. Importantly, the present results derive from controlled intervention studies focusing specifically on dry-cured ham as a distinct food product, rather than on heterogeneous categories of processed meats. The observed neutral-to-modest effects are biologically plausible in light of the specific nutritional and biochemical characteristics of dry-cured ham, including its relatively high monounsaturated fatty acid (MUFA) content and the presence of curing-generated peptides described in the product literature. However, the human intervention studies included in the present review did not directly quantify circulating ham-derived peptides or assess their in vivo activity after consumption. Therefore, any contribution of these peptides to the observed cardiometabolic effects should be regarded as mechanistically plausible but not directly demonstrated. Taken together, these findings reinforce the concept that processed meats are not nutritionally homogeneous. Food-matrix characteristics and processing methods should therefore be taken into account when interpreting epidemiological evidence and formulating dietary recommendations [31,32,33].

### 4.1. Effects of Dry-Cured Ham on Blood Pressure

In the pooled analysis, dry-cured ham consumption did not significantly affect systolic blood pressure but was associated with a small yet statistically significant reduction in diastolic blood pressure of approximately −1.6 mmHg compared with control products. Although the absolute magnitude of this effect is modest at the individual level, small average reductions in diastolic blood pressure have been associated with meaningful decreases in cardiovascular events when considered at the population level [34]. Nevertheless, given the short duration of the included interventions and the substantial between-study heterogeneity, the clinical relevance of this reduction at the individual level should be interpreted with caution. Importantly, none of the included trials reported clinically relevant increases in blood pressure or evidence of sodium-related blood pressure worsening during the ham intervention periods [32].

These findings contrast with previous systematic reviews and meta-analyses of prospective cohort studies reporting a higher incidence of hypertension associated with greater consumption of processed meats. For example, Schwingshackl et al. observed a 12–14% higher risk of hypertension associated with both red and processed meat intake in dose–response analyses [32]. Mechanistic reviews have further highlighted the high sodium [35] and nitrite content [36] of typical processed meats as key mediators of elevated blood pressure and adverse cardiovascular outcomes, noting that processed red meats may contain approximately four-fold more sodium and substantially higher nitrite additive levels than unprocessed meats [37]. In this context, the absence of harmful blood pressure effects observed in the present meta-analysis suggests that the specific formulation and composition of dry-cured Iberian ham, consumed in portions ranging from 40 to 120 g/day in the included trials and characterized by a distinct food matrix, may attenuate mechanisms traditionally implicated in processed meat-related hypertension [32].

The blood pressure results also align with evidence from dietary intervention trials focusing on MUFA-rich diets. A systematic review and meta-analysis of randomized controlled trials comparing high- versus low-MUFA regimens (>12% vs. ≤12% of total energy) reported significant reductions in systolic (−2.3 mmHg) and diastolic (−1.2 mmHg) blood pressure in favor of high-MUFA diets [10]. A broader synopsis of systematic reviews and meta-analyses similarly concluded that MUFA-rich dietary patterns tend to lower blood pressure [38] and improve glycemic control [39] without detrimental effects on blood lipids [11]. Given that dry-cured Iberian ham is relatively rich in oleic acid, these data provide biological plausibility for the modest diastolic blood pressure reduction observed in the present meta-analysis, particularly among individuals with prehypertension or mild cardiometabolic risk.

### 4.2. Effects on Lipid Profile and Cardiometabolic Markers

Regarding lipid parameters, the pooled quantitative synthesis showed a modest but statistically significant reduction in total cholesterol, approximately −5 mg/dL, in the intervention group compared with the control group, whereas no significant overall effects were observed for LDL cholesterol, HDL cholesterol, or triglyceride concentrations. Although individual trials reported potentially favorable changes in specific lipid fractions, including decreases in LDL cholesterol and triglycerides or increases in HDL cholesterol following moderate dry-cured ham consumption, these effects were not consistently confirmed in the pooled analyses. Collectively, these findings indicate that, within the intake ranges and intervention durations examined, dry-cured ham consumption does not adversely affect lipid profiles and may be associated with small reductions in total cholesterol in selected adult populations.

The lipid findings are broadly compatible with evidence from meta-analyses of dietary patterns rich in monounsaturated fatty acids. Schwingshackl et al. [11] summarized long-term randomized controlled trials and reported that high-MUFA interventions tended to improve HDL cholesterol and reduce triacylglycerols, with inconsistent but generally non-adverse effects on total and LDL cholesterol. Another meta-analysis by the same group focusing on cardiovascular risk factors found significant reductions in fat mass and blood pressure with high-MUFA diets, again without evidence of lipid deterioration [10]. More recently, analyses of data from the National Health and Nutrition Examination Survey (NHANES) indicated that higher intakes of monounsaturated and polyunsaturated fatty acids were associated with a lower estimated 10-year risk of major atherosclerotic cardiovascular events [40]. Considering that dry-cured Iberian ham provides relevant amounts of MUFA within a mixed diet, these data support the biological plausibility of a neutral or modestly favorable lipid response, rather than a clear lipid-lowering effect, when consumed in controlled portions.

In contrast, large-scale observational systematic reviews and meta-analyses have generally reported positive associations between processed meat intake and adverse cardiometabolic outcomes. A comprehensive meta-analysis of prospective cohort studies concluded that both unprocessed and processed red meat consumption were associated with higher risks of cardiovascular disease and type 2 diabetes, with somewhat stronger associations observed in Western populations [4]. Similarly, a systematic review and meta-analysis published in the Annals of Internal Medicine reported that reducing processed meat intake by three servings per week was associated with only small reductions in all-cause mortality and cardiometabolic outcomes, although still in a harmful direction [15]. The present intervention-based findings therefore differ from the average associations reported for broadly defined processed meat categories and suggest that dry-cured ham, when consumed in moderate portions as part of an otherwise balanced diet, may not confer the same degree of cardiometabolic risk typically attributed to processed meats in epidemiological research.

### 4.3. Effects on Fasting Blood Glucose and Glycemic Control

The pooled meta-analysis did not demonstrate a statistically significant effect of dry-cured ham consumption on fasting blood glucose compared with control conditions, although point estimates suggested a small, non-significant reduction of approximately 2 mg per dL. These findings indicate a neutral effect on short-term glycemic control within the intake ranges and intervention durations evaluated.

This neutral effect differs from evidence derived from meta-analyses examining dietary fat quality, in which monounsaturated-fatty-acid-rich diets have been associated with improvements in glycemic control, including reductions in fasting blood glucose and glycated hemoglobin, particularly among individuals with type 2 diabetes [11,39,41]. It also contrasts with large-scale cohort meta-analyses reporting a higher risk of type 2 diabetes associated with greater consumption of unprocessed and processed red meat, although these associations are modest in magnitude and based on observational evidence [4,15]. Several factors may explain these apparent discrepancies, including the relatively short duration of the included intervention trials, the predominance of non-diabetic participants with near-normal baseline glycemia, and the consumption of dry-cured ham within mixed meals, in which its specific glycemic impact is likely small relative to overall dietary patterns.

Taken together, the available intervention evidence suggests that moderate dry-cured ham consumption does not adversely affect fasting blood glucose in adults without diabetes. However, longer-term randomized controlled trials in populations with impaired glucose metabolism are required to determine whether dry-cured ham has any clinically relevant effect, either beneficial or harmful, on glycemic regulation.

### 4.4. Interactive Effects and Potential Health Implications

Although the findings of the present review were discussed according to specific outcome domains, it is unlikely that these effects operate in isolation [24,25,26,27,28]. Blood pressure regulation, lipid metabolism, endothelial function, inflammatory activity, and oxidative balance are closely interrelated processes in cardiometabolic health [42]. For this reason, the improvements observed across some of these domains may reflect a broader physiological response rather than a series of unrelated findings [43]. This may be particularly relevant in the case of dry-cured ham, whose nutritional and biochemical characteristics differ from those of many other processed meat products [44].

A plausible interpretation is that the overall effect of dry-cured ham depends on the interaction between components that have traditionally raised concern, such as sodium, and other constituents that may partly modulate the cardiometabolic response [7,35]. In the studies included in this review, dry-cured ham was consumed as a whole food under controlled conditions, and, in that context, it did not appear to worsen conventional cardiometabolic markers [24,25,26,27,28]. By contrast, some trials suggested modestly favorable changes in diastolic blood pressure, total cholesterol, endothelial-related markers, inflammatory mediators, and oxidative stress parameters [24,25,26,27,28]. Although these effects were not uniform across studies, taken together, they support the idea that dry-cured ham should not be interpreted solely on the basis of its sodium content or its classification within the broad category of processed meats [45].

From a broader health perspective, these findings suggest that moderate consumption of dry-cured ham may have a different cardiometabolic profile from that usually attributed to processed meats as a whole [4,45]. This does not imply that dry-cured ham should be regarded as a cardioprotective food, but it does support a more nuanced interpretation in which food matrix, fatty acid composition, curing-related peptide generation, portion size, and overall dietary context are all taken into account [7,46]. Although curing-generated peptides may contribute to this interpretation, their circulating concentrations and in vivo activity were not directly assessed in the included trials. Therefore, the broader implications of these findings should still be considered preliminary, given the limited number of intervention studies and their relatively short duration [24,25,26,27,28]. At present, the available evidence is insufficient to support formal reclassification of dry-cured ham within processed meat categories or to justify revisions to existing dietary guidelines. Rather, the findings highlight the need for future research to determine whether distinctions based on food matrix and processing characteristics should be considered more explicitly in classification frameworks and dietary guidance [47].

### 4.5. Limitations and Future Research Directions

This systematic review and meta-analysis have several limitations that should be considered when interpreting the findings. First, the number of available intervention studies was small, which limited the precision of the pooled estimates and contributed to substantial heterogeneity across several outcomes. Given the variability in study design, comparators, and ham type, formal subgroup analyses or meta-regression were not feasible. Second, most interventions were short-term (4–8 weeks) and evaluated moderate intakes of dry-cured ham (40–120 g/day), which limits inference regarding habitual long-term consumption. Third, although most studies used randomized designs, some concerns remained regarding selective reporting, open-label designs, and limited information on pre-registered protocols, while the two non-randomized studies were judged to be at serious risk of bias due to confounding and the lack of control groups.

Additional limitations affect mechanistic interpretation and external validity. Although bioactive peptides are discussed as a plausible explanation for some observed effects, none of the included trials directly measured circulating ham-derived peptides or their biological activity in vivo.

Moreover, all included trials were conducted in Spain and evaluated specific Iberian dry-cured ham or dry-cured ham products, which may differ from similar products consumed in other settings. The interventions were also heterogeneous in terms of ham type, and no stratified analysis according to product characteristics was possible; therefore, potential differences in health effects between acorn-fed and feed-fed Iberian ham, or between Iberian and serrano ham, cannot be excluded. In addition, the relatively small sample sizes and the focus on intermediate biomarkers, rather than hard clinical endpoints [48], preclude any definitive conclusions regarding long-term cardiovascular disease, type 2 diabetes, or mortality outcomes. Another relevant issue is the potential replacement effect, since in several studies dry-cured ham was introduced within the habitual diet or substituted for other meat products, meaning that its apparent health impact may partly depend on the nutritional profile of the food it replaces.

Future research should prioritize adequately powered, longer-term randomized controlled trials comparing dry-cured ham with well-characterized alternative protein sources or meat products, using standardized portions and detailed product characterization. Such trials should also include populations, assess dose–response relationships, and incorporate vascular, inflammatory, thrombotic, and mechanistic outcomes. In parallel, prospective cohort studies that clearly distinguish dry-cured ham from other processed meats are needed to clarify its long-term cardiometabolic implications.

## 5. Conclusions

This systematic review and meta-analysis summarizes the available evidence from human intervention studies examining the cardiometabolic and vascular effects of dry-cured ham consumption in adults. Overall, the pooled analyses indicated a small but statistically significant reduction in diastolic blood pressure and total cholesterol, while no significant effects were observed for systolic blood pressure, LDL cholesterol, HDL cholesterol, triglycerides, or fasting blood glucose. The magnitude of these effects was modest and was characterized by substantial between-study heterogeneity, which should be considered when interpreting the findings.

Taken together, the current intervention evidence suggests that moderate consumption of Iberian dry-cured ham, when consumed in controlled portions (40–120 g/day), does not appear to adversely influence conventional cardiometabolic risk markers in the studied populations. These findings should be interpreted with caution. The neutral to modest effects observed may be partly related to the specific food matrix of dry-cured ham, including its lipid composition and the generation of bioactive peptides during the curing process.

Even so, the limited number of trials, their short duration, and the reliance on intermediate biomarkers preclude firm conclusions regarding long-term cardiometabolic outcomes. Further well-designed and longer-term randomized controlled trials are needed to better contextualize these findings. Prospective cohort studies that clearly distinguish dry-cured ham from other processed meats will also be important for informing future dietary guidance. At present, however, the current evidence is not sufficient to justify formal reclassification of dry-cured ham or revisions to existing dietary guidelines. Rather, it supports the need for a more differentiated research framework to determine whether such distinctions are warranted.

## Data Availability

No new data were created or analyzed in this study. Data sharing is not applicable.

## Supplementary Materials

The following supporting information can be downloaded at: https://www.mdpi.com/article/10.3390/foods15071198/s1, Figure S1. Sensitivity analyses of the effects of the intervention on lipid profile parameters using an alternative within-subject correlation assumption (r = 0.5). Forest plots are shown for (A) total cholesterol, (B) low-density lipoprotein cholesterol, (C) high-density lipoprotein cholesterol, and (D) triglycerides. Effect estimates are expressed as mean differences with 95% confidence intervals; Figure S2. Sensitivity analyses of the effects of the intervention on blood pressure parameters using an alternative within-subject correlation assumption (r = 0.5). Forest plots are shown for (A) systolic blood pressure and (B) diastolic blood pressure. Effect estimates are expressed as mean differences with 95% confidence intervals; Figure S3. Sensitivity analysis of the effects of the intervention on fasting blood glucose using an alternative within-subject correlation assumption (r = 0.5). Effect estimates are expressed as mean differences with 95% confidence intervals and were pooled using random-effects models. Table S1. Search Strategy. File S1. PRISMA_2020_checklist.

## Abbreviations

The following abbreviations are used in this manuscript:

CVD	Cardiovascular disease
GBD	Global Burden of Disease
HDL	High-density lipoprotein
IL-6	Interleukin-6
LDL	Low-density lipoprotein
MCP-1	Monocyte Chemoattractant Protein-1
MUFA	Monounsaturated fatty acid
PAI-1	Plasminogen activator inhibitor-1

## References

[1] Roth G.A., Mensah G.A., Johnson C.O., Addolorato G., Ammirati E., Baddour L.M., Barengo N.C., Beaton A.Z., Benjamin E.J., Benziger C.P. (2020). Global Burden of Cardiovascular Diseases and Risk Factors, 1990–2019: Update From the GBD 2019 Study. J. Am. Coll. Cardiol..

[2] Afshin A., Sur P.J., Fay K.A., Cornaby L., Ferrara G., Salama J.S., Mullany E.C., Abate K.H., Abbafati C., Abebe Z. (2019). Health Effects of Dietary Risks in 195 Countries, 1990–2017: A Systematic Analysis for the Global Burden of Disease Study 2017. Lancet.

[3] Murray C.J.L., Aravkin A.Y., Zheng P., Abbafati C., Abbas K.M., Abbasi-Kangevari M., Abd-Allah F., Abdelalim A., Abdollahi M., Abdollahpour I. (2020). Global Burden of 87 Risk Factors in 204 Countries and Territories, 1990–2019: A Systematic Analysis for the Global Burden of Disease Study 2019. Lancet.

[4] Shi W., Huang X., Schooling C.M., Zhao J.V. (2023). Red Meat Consumption, Cardiovascular Diseases, and Diabetes: A Systematic Review and Meta-Analysis. Eur. Heart J..

[5] Zhong V.W., Van Horn L., Greenland P., Carnethon M.R., Ning H., Wilkins J.T., Lloyd-Jones D.M., Allen N.B. (2020). Associations of Processed Meat, Unprocessed Red Meat, Poultry, or Fish Intake With Incident Cardiovascular Disease and All-Cause Mortality. JAMA Intern. Med..

[6] Smith S.B., Lunt D.K., Smith D.R., Walzem R.L. (2020). Producing High-Oleic Acid Beef and the Impact of Ground Beef Consumption on Risk Factors for Cardiovascular Disease: A Review. Meat Sci..

[7] Hernández Correas N., Liceaga A.M., Abellán A., Muñoz-Rosique B., Tejada L. (2025). Bioactivities Derived from Dry-Cured Ham Peptides: A Review. Antioxidants.

[8] Gimeno Beviá V., Vandecandelaere E., Marie-Vivien D., Thévenod-Mottet E., Bouhaddane M., Pieprzownik V., Tartanac F., Puzone I. (2025). The Traditional Specialty Guaranteed or the Protected Geographical Indication as Quality Schemes for the Protection of Jamón Serrano (Serrano Ham). Worldwide Perspectives on Geographical Indications: Crossed Views Between Researchers, Policy Makers and Practitioners.

[9] Hu Y., Xiao S., Zhou G., Chen X., Wang B., Wang J. (2024). Bioactive Peptides in Dry-Cured Ham: A Comprehensive Review of Preparation Methods, Metabolic Stability, Safety, Health Benefits, and Regulatory Frameworks. Food Res. Int..

[10] Schwingshackl L., Strasser B., Hoffmann G. (2011). Effects of Monounsaturated Fatty Acids on Cardiovascular Risk Factors: A Systematic Review and Meta-Analysis. Ann. Nutr. Metab..

[11] Schwingshackl L., Hoffmann G. (2012). Monounsaturated Fatty Acids and Risk of Cardiovascular Disease: Synopsis of the Evidence Available from Systematic Reviews and Meta-Analyses. Nutrients.

[12] Di Nunzio M., Loffi C., Montalbano S., Chiarello E., Dellafiora L., Picone G., Antonelli G., Tedeschi T., Buschini A., Capozzi F. (2022). Cleaning the Label of Cured Meat; Effect of the Replacement of Nitrates/Nitrites on Nutrients Bioaccessibility, Peptides Formation, and Cellular Toxicity of In Vitro Digested Salami. Int. J. Mol. Sci..

[13] Andrade B.F., do Carmo L.R., Tanaka M.S., de Almeida Torres Filho R., de Lemos Souza Ramos A., Ramos E.M. (2025). Evaluation of Nonthermal Technologies to Reduce or Replace Nitrite in Meat Products. Food Technol. Biotechnol..

[14] Ferysiuk K., Wójciak K.M. (2020). Reduction of Nitrite in Meat Products through the Application of Various Plant-Based Ingredients. Antioxidants.

[15] Zeraatkar D., Han M.A., Guyatt G.H., Vernooij R.W.M., El Dib R., Cheung K., Milio K., Zworth M., Bartoszko J.J., Valli C. (2019). Red and Processed Meat Consumption and Risk for All-Cause Mortality and Cardiometabolic Outcomes: A Systematic Review and Meta-Analysis of Cohort Studies. Ann. Intern. Med..

[16] Montoro-García S., Zafrilla-Rentero M.P., Celdrán-de Haro F.M., Piñero-de Armas J.J., Toldrá F., Tejada-Portero L., Abellán-Alemán J. (2017). Effects of Dry-Cured Ham Rich in Bioactive Peptides on Cardiovascular Health: A Randomized Controlled Trial. J. Funct. Foods.

[17] Rico-Campà A., Sayón-Orea C., Martínez-González M.Á., Ruiz-Canela M., Ruiz-Estigarribia L., de la Fuente-Arrillaga C., Toledo E., Bes-Rastrollo M. (2020). Cured Ham Consumption and Incidence of Hypertension: The “Seguimiento Universidad de Navarra” (SUN) Cohort. Med. Clín..

[18] Page M.J., McKenzie J.E., Bossuyt P.M., Boutron I., Hoffmann T.C., Mulrow C.D., Shamseer L., Tetzlaff J.M., Akl E.A., Brennan S.E. (2021). The PRISMA 2020 Statement: An Updated Guideline for Reporting Systematic Reviews. BMJ.

[19] McKenzie J.E., Brennan S.E., Ryan R.E., Thomson H.J., Johnston R.V., Thomas J. (2019). Defining the Criteria for Including Studies and How They Will Be Grouped for the Synthesis. Cochrane Handbook for Systematic Reviews of Interventions.

[20] Higgins J.P., Savović J., Page M.J., Elbers R.G., Sterne J.A. (2019). Assessing Risk of Bias in a Randomized Trial. Cochrane Handbook for Systematic Reviews of Interventions.

[21] Sterne J.A.C., Savović J., Page M.J., Elbers R.G., Blencowe N.S., Boutron I., Cates C.J., Cheng H.-Y., Corbett M.S., Eldridge S.M. (2019). RoB 2: A Revised Tool for Assessing Risk of Bias in Randomised Trials. BMJ.

[22] Sterne J.A., Hernán M.A., Reeves B.C., Savović J., Berkman N.D., Viswanathan M., Henry D., Altman D.G., Ansari M.T., Boutron I. (2016). ROBINS-I: A Tool for Assessing Risk of Bias in Non-Randomised Studies of Interventions. BMJ.

[23] Iheozor-Ejiofor Z., Savović J., Bowater R.J., Higgins J.P.T. (2026). The Application of ROBINS-I Guidance in Systematic Reviews of Non-Randomised Studies: A Descriptive Study. Res. Synth. Methods.

[24] Montoro-García S., Velasco-Soria Á., Mora L., Carazo-Díaz C., Prieto-Merino D., Avellaneda A., Miranzo D., Casas-Pina T., Toldrá F., Abellán-Alemán J. (2022). Beneficial Impact of Pork Dry-Cured Ham Consumption on Blood Pressure and Cardiometabolic Markers in Individuals with Cardiovascular Risk. Nutrients.

[25] Martínez-Sánchez S.M., Minguela A., Prieto-Merino D., Zafrilla-Rentero M.P., Abellán-Alemán J., Montoro-García S. (2017). The Effect of Regular Intake of Dry-Cured Ham Rich in Bioactive Peptides on Inflammation, Platelet and Monocyte Activation Markers in Humans. Nutrients.

[26] Mayoral P., Martinez-Salgado C.S., Santiago J.M., Rodriguez-Hernandez M.V., García-Gomez M.L., Morales A., López-Novoa J.M., Macías-Nuñez J.F. (2003). Effect of Ham Protein Substitution on Oxidative Stress in Older Adults. J. Nutr. Health Aging.

[27] Saban-Ruiz J., Fabregate-Fuente M., Fabregate-Fuente R., Andres-Castillo A., Palomino-Antolin A., Barrio-Carreras D., Martin-Fernandez L., Altamirano F., Fernandez-Fernandez C., Andres-Lacueva C. (2017). Iberian Cured-Ham Consumption Improves Endothelial Function in Healthy Subjects. J. Nutr. Health Aging.

[28] Márquez-Contreras E., Vázquez-Rico I., Baldonedo-Suárez A., Márquez-Rivero S., Jiménez J., Machancoses F., Morano-Báez R., León-Justel A. (2018). Effect of Moderate and Regular Consumption of Cinco Jotas Acorn-fed 100% Iberian Ham on Overall Cardiovascular Risk: A Cohort Study. Food Sci. Nutr..

[29] Montoro-Garcia S., Velasco-Soria A., Abellan-Aleman J. (2022). Beneficial Impact of Pork Dry-Cured Ham Consumption on Blood Pressure and Cardiometabolic Markers in Individuals with High Cardiovascular Risk. Cardiovasc. Res..

[30] Mora L., Escudero E., Arihara K., Toldrá F. (2015). Antihypertensive Effect of Peptides Naturally Generated during Iberian Dry-Cured Ham Processing. Food Res. Int..

[31] Touvier M., da Costa Louzada M.L., Mozaffarian D., Baker P., Juul F., Srour B. (2023). Ultra-Processed Foods and Cardiometabolic Health: Public Health Policies to Reduce Consumption Cannot Wait. BMJ.

[32] Schwingshackl L., Schwedhelm C., Hoffmann G., Knüppel S., Iqbal K., Andriolo V., Bechthold A., Schlesinger S., Boeing H. (2017). Food Groups and Risk of Hypertension: A Systematic Review and Dose-Response Meta-Analysis of Prospective Studies. Adv. Nutr..

[33] Fardet A., Rock E. (2019). Ultra-Processed Foods: A New Holistic Paradigm?. Trends Food Sci. Technol..

[34] Rahimi K., Bidel Z., Nazarzadeh M., Copland E., Canoy D., Wamil M., Majert J., McManus R., Adler A., Agodoa L. (2021). Age-Stratified and Blood-Pressure-Stratified Effects of Blood-Pressure-Lowering Pharmacotherapy for the Prevention of Cardiovascular Disease and Death: An Individual Participant-Level Data Meta-Analysis. Lancet.

[35] Kong F., Liu Q., Zhou Q., Xiao P., Bai Y., Wu T., Xia L. (2025). Dietary Salt Intake and Cardiovascular Outcomes: An Umbrella Review of Meta-Analyses and Dose-Response Evidence. Ann. Med..

[36] Norouzzadeh M., Hasan Rashedi M., Payandeh N., Mirdar Harijani A., Shahinfar H. (2024). The Effects of Dietary Nitrate on Blood Pressure and Vascular Health: An Umbrella Review and Updated Meta-Analysis and Meta-Regression. J. Funct. Foods.

[37] Allen T.S., Bhatia H.S., Wood A.C., Momin S.R., Allison M.A. (2022). State-of-the-Art Review: Evidence on Red Meat Consumption and Hypertension Outcomes. Am. J. Hypertens..

[38] Zang T., Hassan W., Javaid F., Shabbir R., Shahzadi A., Ahmed H. (2025). Impact of Fatty Diets on Blood Pressure: A Systematic Review and Meta-Analysis. Asia Pac. J. Clin. Nutr..

[39] Sanz-París A., Matía-Martín P., Martín-Palmero Á., Gómez-Candela C., Camprubi Robles M. (2020). Diabetes-Specific Formulas High in Monounsaturated Fatty Acids and Metabolic Outcomes in Patients with Diabetes or Hyperglycaemia. A Systematic Review and Meta-Analysis. Clin. Nutr..

[40] Yang Z., Yang K., Zhang X., Yang Q., Zhang Y., Gao J., Qu H., Shi J. (2023). Dietary Saturated, Monounsaturated, or Polyunsaturated Fatty Acids and Estimated 10-Year Risk of a First Hard Cardiovascular Event. Am. J. Med..

[41] Qian F., Korat A.A., Malik V., Hu F.B. (2016). Metabolic Effects of Monounsaturated Fatty Acid-Enriched Diets Compared With Carbohydrate or Polyunsaturated Fatty Acid-Enriched Diets in Patients with Type 2 Diabetes: A Systematic Review and Meta-Analysis of Randomized Controlled Trials. Diabetes Care.

[42] Incalza M.A., D’Oria R., Natalicchio A., Perrini S., Laviola L., Giorgino F. (2018). Oxidative Stress and Reactive Oxygen Species in Endothelial Dysfunction Associated with Cardiovascular and Metabolic Diseases. Vasc. Pharmacol..

[43] Valaitienė J., Laučytė-Cibulskienė A. (2024). Oxidative Stress and Its Biomarkers in Cardiovascular Diseases. Artery Res..

[44] Miller G.D., Ragalie-Carr J., Torres-Gonzalez M. (2023). Perspective: Seeing the Forest Through the Trees: The Importance of Food Matrix in Diet Quality and Human Health. Adv. Nutr..

[45] De Smet S., Van Hecke T. (2024). Meat Products in Human Nutrition and Health—About Hazards and Risks. Meat Sci..

[46] Aguilera J.M. (2025). Food Matrices as Delivery Units of Nutrients in Processed Foods. J. Food Sci..

[47] Trumbo P.R., Bleiweiss-Sande R., Campbell J.K., Decker E., Drewnowski A., Erdman J.W., Ferruzzi M.G., Forde C.G., Gibney M.J., Hess J.M. (2024). Toward a Science-Based Classification of Processed Foods to Support Meaningful Research and Effective Health Policies. Front. Nutr..

[48] Fleming T.R., Powers J.H. (2012). Biomarkers and Surrogate Endpoints In Clinical Trials. Stat. Med..

