# Supplementary material for: Effects of Dry-Cured Ham Consumption on Cardiometabolic and Vascular Health in Adults: A Systematic Review and Meta-Analysis of Human Intervention Studies
Source: Foods. 2026 Apr 2;15(7):1198. doi: 10.3390/foods15071198 (PMC13073582; doi:10.3390/foods15071198)
Supplement: Supplementary file 1 [file foods-15-01198-s001.zip › foods-4212708-supplementary/Table S1. Search strategy.pdf]

Table S1. Search strategy.

**PubMed/Medline**: February 19, 2026 (34)
("cured ham"[Title/Abstract] OR "dry-cured ham"[Title/Abstract] OR "Iberian ham"[Title/Abstract] OR "Spanish ham"[Title/Abstract]) AND ("blood pressure"[Title/Abstract] OR hypertension[Title/Abstract] OR "ambulatory blood pressure"[Title/Abstract] OR "lipid profile"[Title/Abstract] OR cholesterol[Title/Abstract] OR LDL[Title/Abstract] OR triglycerides[Title/Abstract] OR "cardiovascular risk"[Title/Abstract] OR inflammation[Title/Abstract] OR "oxidative stress"[Title/Abstract] OR endothelial[Title/Abstract])
**Cochrane**: February 19, 2026 (15)
("cured ham" OR "dry-cured ham" OR "Iberian ham" OR "Spanish ham" OR "jamón ibérico" OR "jamón curado")
**Scopus**: February 19, 2026 (72)
TITLE-ABS-KEY (("cured ham" OR "dry-cured ham" OR "Iberian ham" OR "Spanish ham" OR "jamón ibérico" OR "jamón curado") AND ("blood pressure" OR hypertension OR "ambulatory blood pressure" OR "lipid profile" OR cholesterol OR LDL OR triglycerides OR "cardiovascular risk" OR inflammation OR "oxidative stress" OR endothelial))
**Web of Science**: February 19, 2026 (152)
TS=(("cured ham" OR "dry-cured ham" OR "Iberian ham" OR "Spanish ham" OR "jamón ibérico" OR "jamón curado") AND ("blood pressure" OR hypertension OR "ambulatory blood pressure" OR "lipid profile" OR cholesterol OR LDL OR triglycerides OR "cardiovascular risk" OR inflammation OR "oxidative stress" OR endothelial))
**Total**: 273
