# Supplementary figures and images for: Effects of Dry-Cured Ham Consumption on Cardiometabolic and Vascular Health in Adults: A Systematic Review and Meta-Analysis of Human Intervention Studies
Source: Foods. 2026 Apr 2;15(7):1198. doi: 10.3390/foods15071198 (PMC13073582; doi:10.3390/foods15071198)

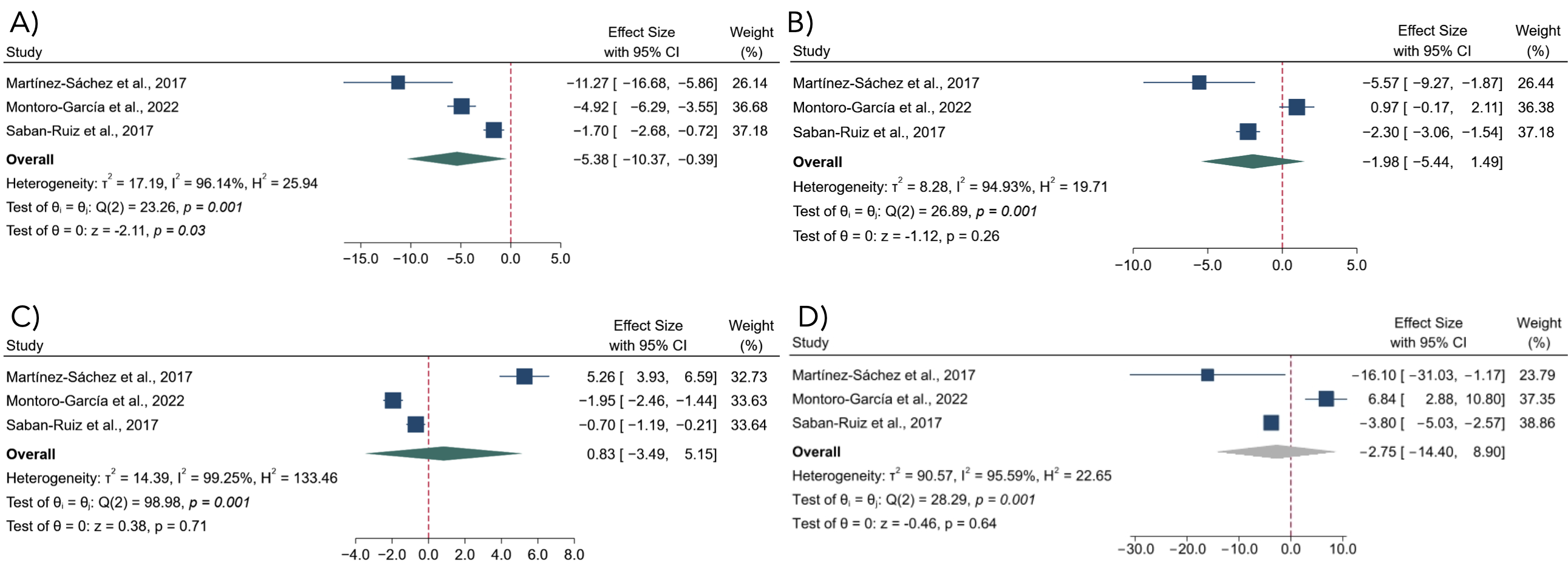

Supplement: Supplementary file 1 [file foods-15-01198-s001.zip › foods-4212708-supplementary/Figure S1.png]

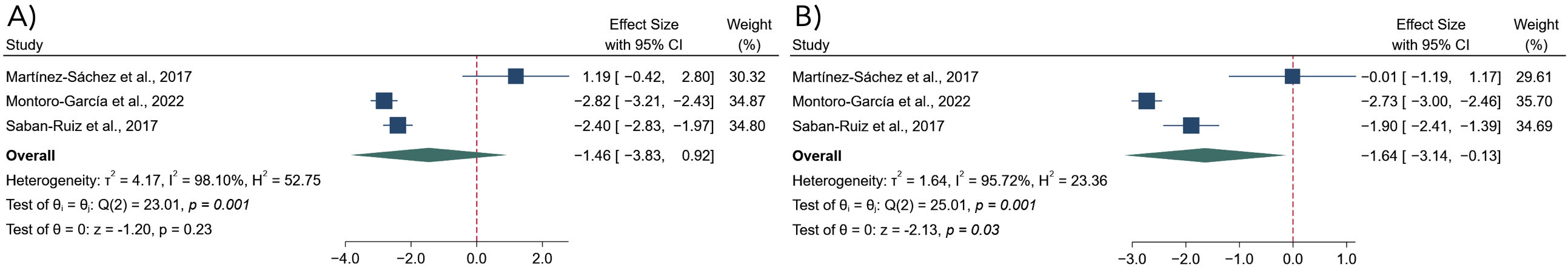

Supplement: Supplementary file 1 [file foods-15-01198-s001.zip › foods-4212708-supplementary/Figure S2.png]

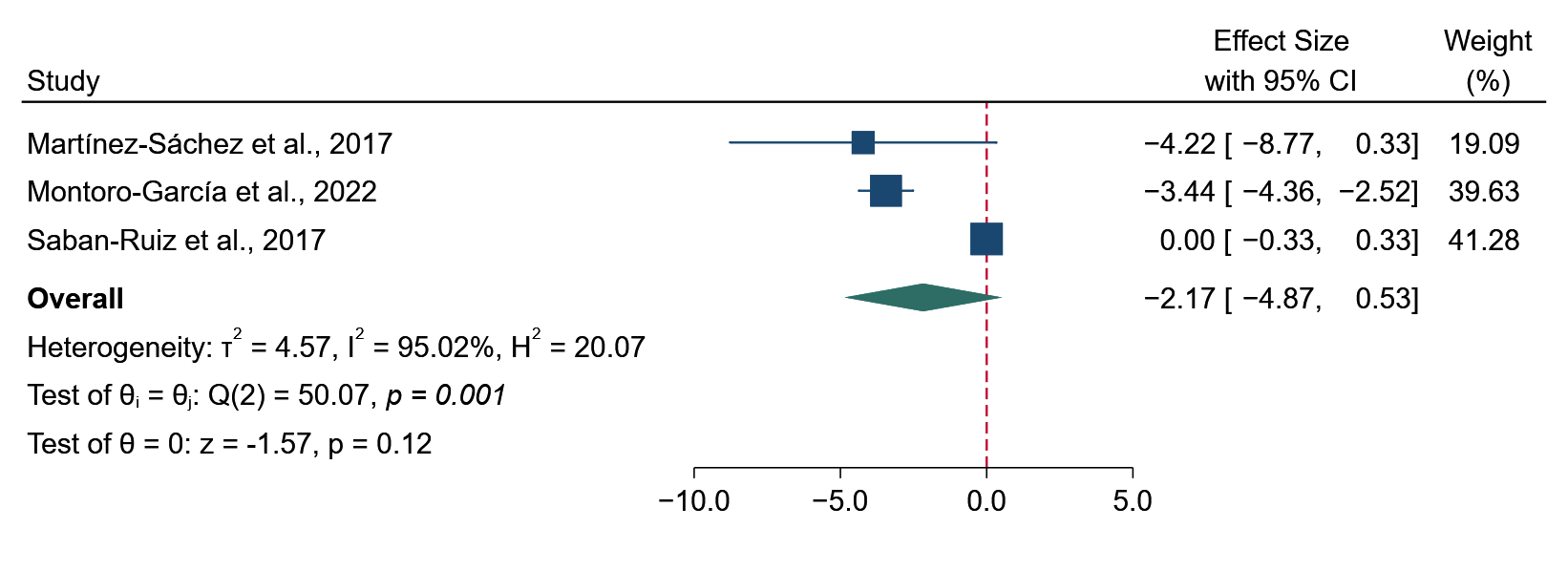

Supplement: Supplementary file 1 [file foods-15-01198-s001.zip › foods-4212708-supplementary/Figure S3.tif]
